# Impact of substrate elasticity on contact angle saturation in electrowetting†

**DOI:** 10.1039/d0sm02281k

**Published:** 2021-03-10

**Authors:** Ioannis E. Markodimitrakis, Dionysios G. Sema, Nikolaos T. Chamakos, Periklis Papadopoulos, Athanasios G. Papathanasiou

**Affiliations:** School of Chemical Engineering, National Technical University of Athens 15780 Greece pathan@chemeng.ntua.gr +30 210 772 3234; Department of Physics, University of Ioannina Greece; Institute of Materials Science and Computing, University Research Center of Ioannina Greece

## Abstract

The electrostatically assisted wettability enhancement of dielectric solid surfaces, commonly termed as electrowetting-on-dielectric (EWOD), facilitates many microfluidic applications due to simplicity and energy efficiency. The application of a voltage difference between a conductive droplet and an insulated electrode substrate, where the droplet sits, is enough for realizing a considerable contact angle change. The contact angle modification is fast and almost reversible; however it is limited by the well-known saturation phenomenon which sets in at sufficiently high voltages. In this work, we experimentally show and computationally support the effect of elasticity and thickness of the dielectric on the onset of contact angle saturation. We found that the effect of elasticity is important especially for dielectric thickness smaller than 10 μm and becomes negligible for thickness above 20 μm. We attribute our findings on the effect of the dielectric thickness on the electric field, as well as on the induced electric stresses distribution, in the vicinity of the three phase contact line. Electric field and electric stresses distribution are numerically computed and support our findings which are of significant importance for the design of soft materials based microfluidic devices.

## Abstract 2

Soft and thin dielectrics favor contact angle saturation in electrowetting. Experiments are supported by detailed numerical computations of the electric field and stress distribution.

## Graphical abstract

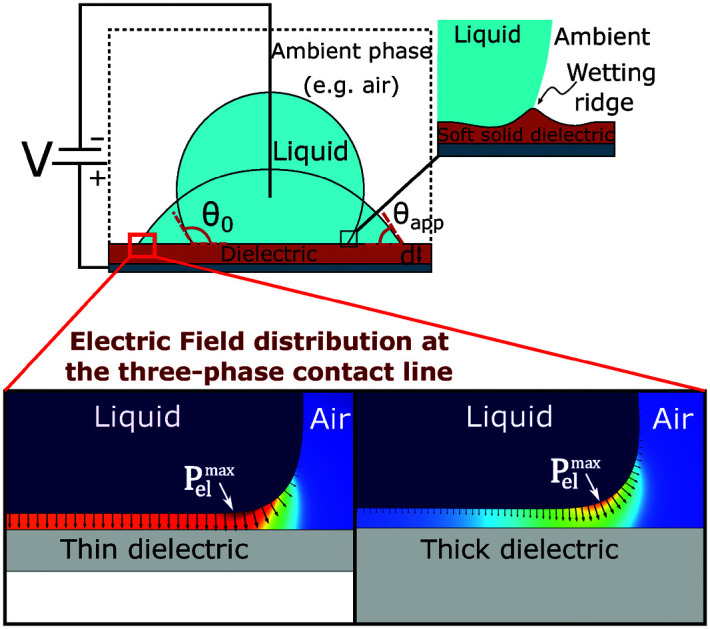

## 1 Introduction

Electrowetting-on-dielectric (EWOD) is already established as a key methodology for active control of the apparent wettability^1^ and mobility of liquid droplets.^2,3^ Electrowetting, *i.e.* the electrostatic enhancement of solid wettability, has been utilized in technological applications such as lab-on-a-chip devices,^4–6^ optofluidic displays^7,8^ as well as liquid lenses^9,10^ and energy harvesting systems.^11,12^ In a typical EWOD configuration, the application of an electric voltage difference between an insulated metal substrate (a flat metal electrode coated by a thin dielectric layer) and a conductive liquid mass, *e.g.* droplets, introduces an excess of electrostatic energy. The electrostatic energy, stored in the charged liquid/insulated solid interface, favors increase of the total capacitance of the system and the spreading of the liquid, manifested as a reduction of the apparent contact angle.^13^

The dependence of the apparent contact angle, *θ*_app_, on the applied voltage, *V*, is stated by the electrowetting (EW) equation (also known as Lippmann equation):^13^1
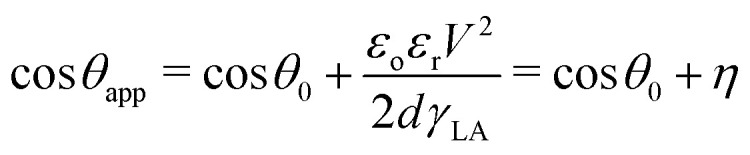
where *θ*_0_ is the initial contact angle (commonly Young's angle) and γ_LA_ is the liquid/ambient interfacial tension. *ε*_o_ and *ε*_r_ stand for the permittivity of vacuum and the dielectric constant of the insulating layer, while, *d*, denotes its thickness. The latter term, *η*, on the right-hand side of eqn (1) is the so-called dimensionless electrowetting number that expresses the relative strength of electrostatic compared to surface tension forces. The Lippmann equation can be derived by minimizing the total energy of the system. From this perspective, the dependence of the contact angle on voltage can also be examined in terms of electromechanical forces that are developed on the liquid surface, by assuming a uniform electric field at the solid/liquid interface and by neglecting the fringe fields near the contact line.^14^

The Lippmann equation has been proven accurate for a variety of EW configurations when the applied voltage is low (commonly for *η* < 0.7). In the case, however, when the applied voltage surpasses a threshold value, the apparent contact angle “deviates” from eqn (1). In particular, contrary to Lippmann's prediction indicating complete wetting at sufficiently high voltage, the reduction of the apparent angle reaches a limit; the limiting phenomenon is widely known as contact angle saturation.^14–16^ Contact angle saturation sets a serious limitation in any EW-based application.^14^

Several theories have been proposed for the mechanism of saturation, however a unified theory is still missing. The most widely accepted argument is that saturation originates from the divergent electric field near the three-phase contact line (TPL). The wedge-like shape of a droplet near the TPL induces singularities of the electric field which may cause nonlinear material responses at high applied voltages.^17,18^ The list of the proposed theories includes charge trapping in the insulating layer,^19–21^ ambient phase ionization^22^ or liquid instabilities, such as micro-droplet ejection.^22,23^

An interesting aspect of this phenomenon has been highlighted by Chevalliot *et al.*^24^ In their work, it was experimentally shown that the final (saturated) contact angle is invariant to several parameters. In particular, the saturation angle was reported to be independent of the thickness, type of the dielectric, surface tension, ionic content of the liquid, or even ambient phase.^24^ Thus, the lowest apparent contact angle that can be achieved does not depend on the specific experimental configuration or materials involved. Saturation usually sets in at approximately 60–80° independent of the surrounding medium (air or oil). Other groups have reported lower saturation angles of about 15–30° using ionic liquids^25^ or glycols,^17^ however, these results have no impact on the fact that the limitation of the electrostatic wettability enhancement is universal for any EWOD configuration.

**Fig. 1:**
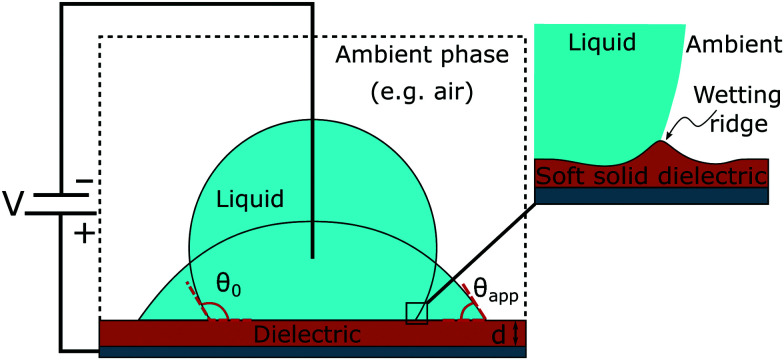
Schematic of a typical EWOD setup. A liquid droplet exhibits an apparent contact angle equal to the initial angle, θ_0_, at zero voltage that is reduced when voltage is applied. Inset: Schematic of electrowetting on a soft dielectric layer and the formation of the wetting ridge near the TPL (not to scale). The size of the ridge is in the order of ∼γ/E, where *E* is the elastic modulus of the dielectric layer.^34^

Several applications, such as microfluidics, involve soft substrates. Recent advances in soft electrowetting^26–29^ raise the question of testing whether the saturation angle shows a similar invariance for deformable substrates. The combination of bulk elasticity and capillary forces on deformable solid surfaces has generated a plethora of observations that seem to deviate from the classical understanding of wetting,^30–34^ like the Shuttleworth effect^31,32^ or the slower spreading dynamics.^30,34^ Elastic substrates are deformed by capillary adhesion forming a wetting ridge near the three-phase contact line (Fig. 1 inset). In the case of a sub millimetric drop on a soft substrate, the formed wetting ridge can reach a height of a few microns that leads to a rise of the TPL and relocation of the local force balance.^30–34^ As the droplet spreads, the motion of the contact line is accompanied by the displacement of the formed ridge, which induces further energy dissipation to the system.^30,34^ Recent evidence^26–29^ has confirmed that the viscoelasticity of the substrate is a primary factor in soft electrowetting that resists droplet spreading. The variation of the elastic modulus of a given type of dielectric can affect the contact angle modification.^27^ In a recent work, the shape of the wetting ridge was also explored using confocal microscopy^29^ in order to find an explanation for the observed deviations.^26,27^ Contrary to expectations, the shape and height of the ridge remained unaffected by the application of voltage. However, the electrowetting number, η, was less than 0.3. Observations on the saturation phenomenon have not been reported up to now.

While the elasticity determines the extent of deformation of the dielectric substrate due to the developed stresses, the dielectric thickness strongly affects the electric field and thus the electric stresses distribution, especially near the TPL. Chamakos *et al.*^35^ have computationally shown that the electrostatic forces on a thick dielectric (*d* > 150 μm) extend beyond the solid/liquid interface to the liquid/ambient interface, at a length scale proportional to the dielectric thickness (*d*). In contrast, for thinner dielectrics (*d* < 10 μm), the field distribution is sharper and the electrical stress is focused at the vicinity of the TPL, explaining the deviation of the microscopic contact angle from Youngs angle that is observed in electrowetting experiments on very thin insulators.^36^ These studies have shown that the liquid profile at the TPL highly sharpens due to high intensification of the electrostatic stress at that region, as the dielectric thickness decreases to less than a few tens of microns. For a relatively thin dielectric layer (*d* ≈ 10 μm), the local curvature of the liquid profile at the TPL increases linearly with the applied voltage, while for a thicker dielectric (*d* ≈ 150 μm), curvature remains virtually unaffected by the voltage.^35^

Here, we study the effects of both thickness and elasticity of the dielectric layer on the contact angle saturation. We conduct electrowetting experiments on poly(dimethyl siloxane) (PDMS) elastomer films with varying Youngs modulus, up to the saturation regime *i.e.* electrowetting numbers, η ≈ 1. In addition, based on our previous computational and experimental studies,^35,37,38^ we calculate the electric stress distribution along the TPL. The range of PDMS thickness is chosen in order to highlight the effect of varying electric stress distribution, and our study is guided by previously performed experimental^36^ and computational^35^ results. Furthermore, we use two liquids, water and propylene glycol, to extend the validity of our observations.

## 2 Material and methods

### Fabrication

Crosslinked polydimethylsiloxane (PDMS) films were prepared on 20 × 20 mm^2^ glass slides, covered with an indium-tin oxide (ITO) layer with the conductivity of 6 Ω cm^−2^ (Fig. S1 in the ESI†). The glass slides were first cleaned with plasma (air, 0.1 mbar, 5 s) and then hydrophobized with hexamethyldisilazane to improve adhesion of the PDMS layer. The elastic modulus of the crosslinked PDMS has been determined by using the Sylgard 184 silicone elastomer kit (Sigma-Aldrich) with varying proportions of monomer to curing agent (Table S1 in the ESI†). The monomer/curing agent mixture was degassed and applied to the glass slides by spin coating at varying speed and duration. All samples were then cured at 60 °C for 20 h. Uncrosslinked molecules were extracted by immersing the samples in ethanol for 24 h and finally the samples were dried in a vacuum for 12 h. The final thickness was estimated by weighing the samples before and after the application of PDMS, with a Mettler Toledo precision balance (0.1 mg), using the density of 0.97 g cm^−3^. The elastic/Youngs modulus is obtained from the literature.^39,40^

### Experimental setup and methods

Contact angle and hysteresis measurements are performed on a commercial goniometer/tensiometer (ramé-hart model 100) equipped with an automated dispensing system. Contact angle hysteresis (*i.e.* the difference between advancing and receding angles of a droplet) is determined by the adding/removing volume method. A droplet of 7 μL is deposited *via* the micro syringe of the dispensing system and rests for 20 s on the sample. With the micro syringe immersed in the drop, its volume is then increased in steps of 0.1 μL s^−1^. The advancing contact angle is measured after each volume increment as the volume increases up to 12 μL. The receding contact angle was measured by decreasing the volume of the droplet with the same rate to a final volume of 2 μL.

The electrowetting experiments are carried out in a classical EWOD setup as depicted in Fig. 1 using water and propylene glycol sessile droplets with mineral salts. In these experiments, DC driving voltages were applied by employing a high-voltage DC-amplifier (EMCO model 4200). A droplet of 10 ± 1 μL is gently pipetted on the examined sample surface. The applied voltage increases step wisely, up to the saturation onset. In each step, the droplet rests for 2.5 s and its shape is analyzed by using the DROPImage Advanced software (provided by ramé-hart). For each data set, at least four electrowetting cycles were performed.

## 3 Results and discussion

Electrowetting experiments were performed on dielectric films of different elasticity and thickness. We used elastomer films with an elastic modulus of 1.7 MPa, 500 kPa, 100 kPa and 40 kPa, as well as three dielectric thickness cases (50 μm, 20 μm and 7 μm).

It is necessary here to clarify that the elasticity of the substrate affects the contact angle hysteresis, the static and the dynamic one. Soft substrates, like the soft elastomer examined in this work, can be substantially deformed under the capillary action, forming a wetting ridge near the TPL.^34^ This ridge acts as a pinning site and affects the macroscopic contact angle and spreading dynamics.^30–34^ Our static contact angle and hysteresis measurements accord with the latter observations showing that the advancing apparent contact angle and the hysteresis increase as the substrates' elastic modulus decreases. It is assumed that the ratio of the monomer to the curing agent of the films under study does not affect the surface chemistry, *i.e.* the material intrinsic wettability (see the ESI†). Therefore, the increase of the apparent contact angle measured is solely due to substrate elasticity. Tables, graphs and more information supporting these measurements can be found in the ESI.† In the main text, all values shown are advancing apparent contact angles, unless otherwise stated.

**Fig. 2:**
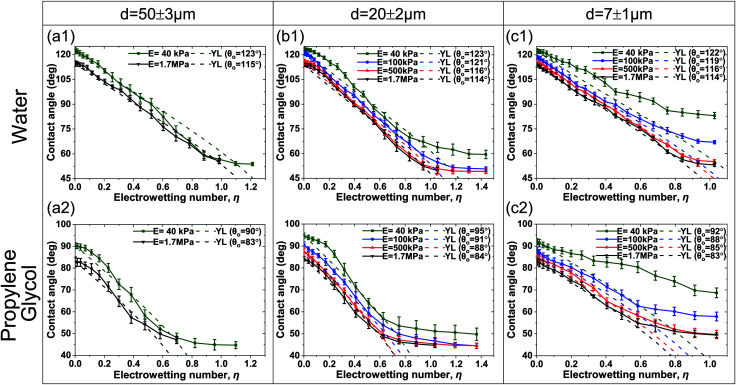
Dependence of the apparent contact angle on the applied voltage for (a) 50 ± 3 μm, (b) 20 ± 2 μm and (c) 7 ± 1 μm thick films. Electrowetting experiments were conducted with (first row) water (γ_LA_ = 0.072 N m^−1^) and (second row) propylene glycol (γ_LA_ = 0.041 N m^−1^). The Lippmann equation is plotted for each corresponding initial contact angle, θ_0_, with a thickness of 50 μm, 20 μm and 7 μm, respectively (ε_o_ = 8.854 × 10^−12^ F m^−1^, ε_r_ = 2.65). (The discontinuity observed in (a) for the hardest dielectric at high voltages is a result of lateral movement of the contact line).

In what follows electrowetting experiments are presented. In the first case, electrowetting experiments were conducted on 50 μm thick elastomer films, the thickest dielectric in this work. In this set of experiments, two films were examined, (a) a rigid-like and (b) a soft film with a Youngs modulus of 1.7 MPa and 40 kPa, respectively. The latter values are the extreme values of elasticity under study. In Fig. 2a, we present the dependence of the apparent contact angle on the applied voltage. We observe that the initial contact angle (for *V* = 0) is higher at the softest dielectric. This difference, initially almost 10°, is clearly visible at low electrowetting numbers, η, though it gets smaller and almost vanishes at η = 0.75, at the saturation regime. This trend is observed for both liquids tested, water and propylene glycol. The saturation angle is ≈54° (for water) and ≈46° (for propylene glycol), respectively, independent of the elasticity of the substrate (see Fig. 2a).

In contrast, elasticity starts to play a role on a thinner (*d* = 7 μm) PDMS substrate. In Fig. 2c, measurements for two additional intermediate elasticities are presented, namely for 500 kPa and 100 kPa. Therein it is shown that the contact angle difference between the softest and the hardest substrates is maintained, even in the saturation regime. In particular, the hardest dielectric shows similar contact angles at saturation as in the previous case (with dielectric thickness 50 μm), whereas in the case of the softest dielectric, saturation is reached at considerably larger angles (82° and 70° for water and propylene glycol, respectively). Interestingly enough, a film with the intermediate elasticity of 100 kPa reached saturation at angles between those of the softest and the hardest dielectric (*i.e.* 68° for water and 59° for propylene glycol), whereas the film with *E* = 500 kPa reached a saturation angle similar to the hardest dielectric. So, it seems that for a given dielectric thickness, *i.e. d* = 7 μm, there is a certain elasticity value, *E*, where the saturation angle starts to deviate from one of the hard substrates.

Similarly, for a given elasticity value, *E*, one can find a thickness value, *d*, that affects the saturation angle. In Fig. 2b, we show the results for an intermediate thickness of *d* = 20 μm. Once again, the initial apparent contact angle values (for *V* = 0) show a slight increase with decreasing elasticity. Here, only the softest sample (*E* = 40 kPa) demonstrates a saturation angle slightly different from that of the rest of the samples studied, ≈60° for water and ≈51° for propylene glycol. Compared to the thinner film (*d* = 7 μm, Fig. 2c), it can be seen that the contact angle difference between the two extreme elasticity cases (*E* = 1.7 MPa and *E* = 40 kPa) is much smaller. This behavior reveals that for this given dielectric thickness, the effect of elasticity is not so pronounced.

Considering the applicability of the Lippmann equation to elastic dielectrics, we observed that its predictions are accurate enough for thick dielectrics, regardless of the elastic modulus, but deviate from the experimental measurements on thin, soft, ones. The thin (*d* = 7 μm), but rigid-like substrates (*E* = 1.7 MPa and 500 kPa) are well addressed, but not the two low-elasticity substrates (*E* ≤ 100 kPa) of the same thickness. More specifically, the 7 μm thick soft film with an elastic modulus of 100 kPa shows a slight deviation from the prediction of Lippmann, above η ≈ 0.55–0.6 (see the ESI†). For the softest film (*E* = 40 kPa), there is a significant deviation even at low voltages (see Fig. 2c).

**Fig. 3:**
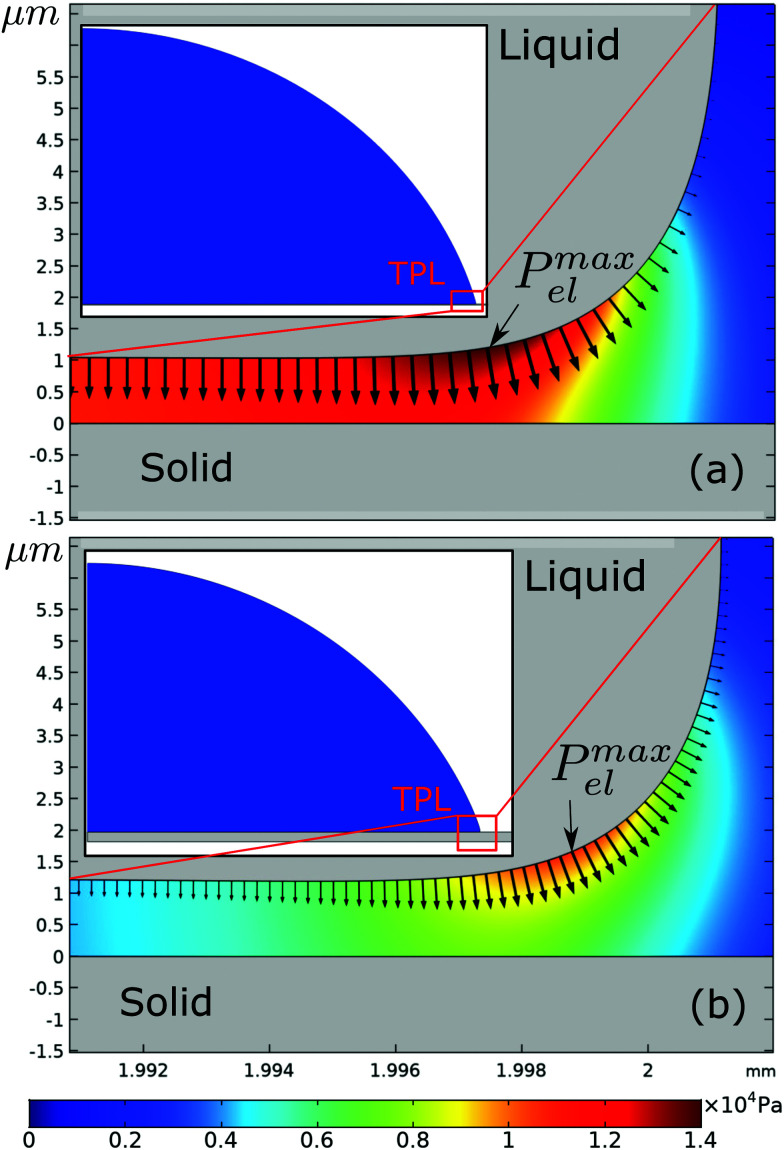
Electrostatic pressure distribution, P_el_, in the ambient phase and along the droplets free surface for the different dielectric cases (a) *d* = 5 μm and (b) *d* = 50 μm (η = 0.8). The arrows follow the electric field vector (are normal to the droplets free surface) and correspond to the directionality of the electrostatic pressure distribution along the liquid-ambient interface at the three-phase contact line (TPL).

In order to gain more insight on the effect of the dielectric thickness and elasticity on the contact angle saturation, we calculated the shape of the drop and the distribution of the electric field near the TPL using a continuum-level computational methodology that was proposed by our group,^41,42^ as well as employed by other researchers.^43^ The shape of entire droplets results from the equilibrium of electrostatic and interfacial forces. Special attention is given at the coupling of the electric field distribution and the solid/liquid or liquid/ambient interfacial shape. In our formulation, we consider that the liquid and the solid phases are separated by an intermediate layer (with thickness δ_min_) stabilized by the presence of the disjoining pressure (see Fig. 3 and the ESI†). The thickness, δ_min_, is a model parameter and is chosen to be sufficiently small as compared to other system dimensions. In particular, when the distance between the solid and and liquid, δ, is equal to δ_min_, the repulsive (steric forces and electrostatic interactions) and attractive forces (van der Waals interactions) counterbalance each other.^41,42^

The computation of the electric field distribution enabled the computation of the distribution of electric stresses at the vicinity of the TPL. For simplicity, the substrate is considered to be rigid and flat, *i.e.* no deformation of the solid is considered. The full coupling of the stress and solid deformation is intended for the following study. The details of the numerical model are presented in the ESI.†

We performed computations for thicknesses, *d*, ranging between 5 μm and 50 μm. In Fig. 3, the electric stress distribution, 
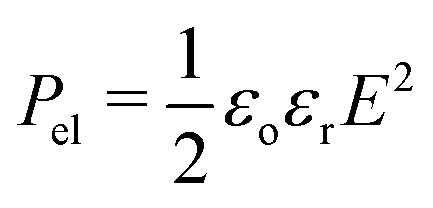
, is shown for *d* = 5 μm and *d* = 50 μm respectively, and for η = 0.8. The model predicts a curved liquid interface near the TPL, which resembles the interface at a liquid/wetting ridge interface (Fig. 1b). The electric stress is always normal to the conducting liquid interface. It is evident that the maximum of the electric stress, as indicated by the color map, is located at the vicinity of the TPL. However, with a careful look, one can see that the maximum is located on the right of the horizontal part of the liquid interface for the case of the thin dielectric (*d* = 5 μm), thus the vector of the maximum stress points almost vertically. For the case of the thick dielectric (*d* = 50 μm), however, the maximum stress is located clearly in part of the interface that is not horizontal (see Fig. 3b). The vector of the maximum stress has a horizontal component which is not negligible. This is the component of the electric stresses that makes the droplet spread, decreasing the (macroscopic) contact angle. The model predicts an apparent contact angle of ≈70 degrees for both thin and thick films compared to the 65 degrees predicted by the Lippmann equation.

**Fig. 4:**
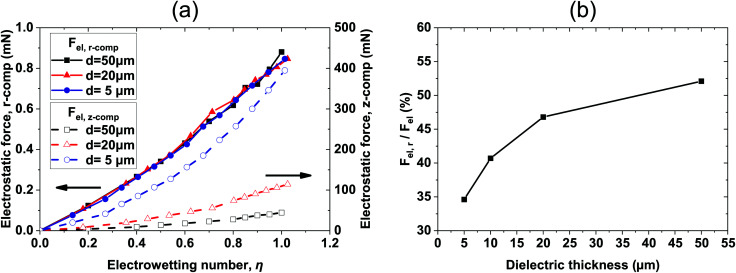
(a) Effective horizontal (read in the left *y*-axis) and vertical (read in the right *y*-axis) component of electrostatic force, F_el_, calculated over the entire surface of the droplet, as a function of the electrowetting number, η. (b) The ratio of the horizontal component of the electrostatic force over the total electric force, F_elr_/F_el_ (%), calculated at the vicinity of the TPL, for selected values of dielectric thickness, *d*, for η = 0.8. (Arrows are placed in (a) as a guide to the eye).

**Fig. 5:**
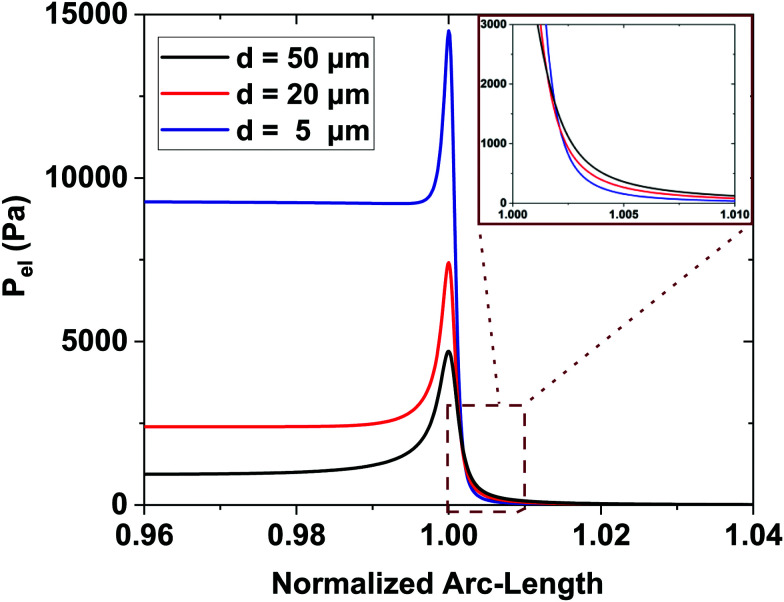
Electrostatic pressure distribution along the droplets free surface in the vicinity of the three-phase contact line for different dielectric thicknesses, and for η = 0.8. The horizontal part at the left side corresponds to the solid/liquid interface, the peak corresponds to the TPL, and the right part corresponds to the liquid/ambient interface, respectively. (A local magnification of the right part of the curves is shown in the inset).

We, then, evaluated the total horizontal, F_elr_, and total vertical, F_elz_, component of the electrostatic force, derived from the electric stress distribution, P_el_, over the entire surface of the droplet. The corresponding quantities read as: 
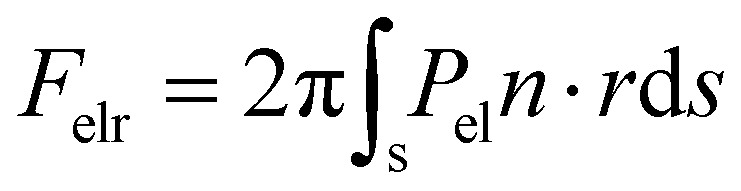
, and 
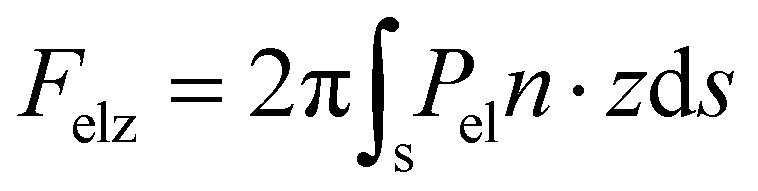
, respectively, where, *n*, is the unit vector normal to the droplets free surface, and, *r*, and, *z*, are the unit vectors in the radial and vertical direction, respectively.^38^ The integral of the radial component of the Maxwell stress, F_elr_, over the liquid surface represents a macroscopic radial force favoring droplet spreading. The dependence of the macroscopic radial and vertical forces on the electrowetting number, η, for selected dielectric thicknesses, *d*, ranging from 5 μm to 50 μm is shown in Fig. 4a. As expected, the macroscopic forces, radial or vertical, increase with η. In particular, the radial component is computed to be independent of the thickness of the dielectric (data shown in Fig. 4a, left axis), when η is fixed. This is in line with the predictions of the Lippmann's equation which wraps up the electrowetting force with the electrowetting number, η. However, the vertical component of the electric force (see data shown in Fig. 4a, right axis) is computed to depend on the thickness, for fixed, η. And the dependence is weak for thick dielectrics, namely, for *d* = 50 μm and *d* = 20 μm, however it is strong for *d* = 5 μm. Thus, the thinner the dielectric the stronger the vertical component of the electric force. Another interesting aspect of the force distribution in the vicinity of the TPL is the ratio of the total electrostatic force responsible for droplet spreading, *i.e.* the magnitude of the horizontal component over the total electrostatic force. This ratio defined as, 
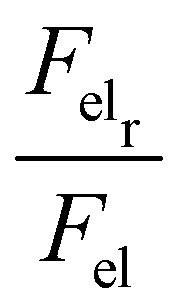
, is calculated at the TPL for various thicknesses (see Fig. 4b). We found that 
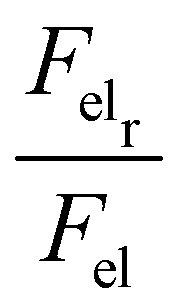
 sharply increases for thicknesses up to *d* = 20 μm. This tendency appears to flatten for higher thicknesses up to *d* = 50 μm, as shown in Fig. 4b.2
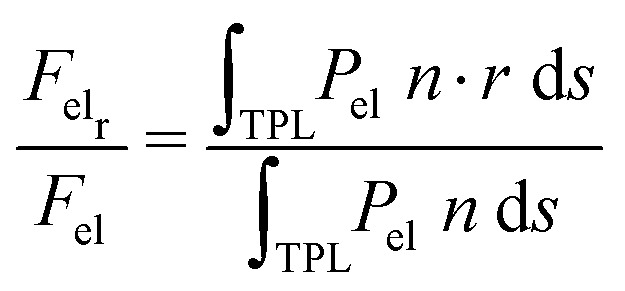
Interestingly enough, the ratio, F_elr_/F_el_, for *d* = 50 μm is 1.5 times the one that corresponds to *d* = 5 μm. In Fig. 5, the electrostatic pressure distribution in the vicinity of the contact line is once again presented, for three dielectric thickness cases, and for η = 0.8. As expected, the electrostatic pressure peaks at the contact line. On the left of the peak, the almost horizontal part of each curve corresponds to the uniform electrostatic pressure at the solid/liquid interface. The direction of the electrostatic pressure is practically vertical there. And the magnitude is higher for the thinnest dielectric studied (i.e. for *d* = 5 μm). A careful look at the three distributions reveals that the electrostatic pressure is highly focused around the TPL and in particular for the case of the thin dielectric. The thicker the dielectric the wider the peak form, supporting that pinning could be stronger for thin dielectrics.

As a consequence, the thickness of the dielectric strongly affects the force balance in the vicinity of the three-phase contact line, where the capillary and elastocapillary forces compete with their electrostatic counterparts. When highly elastic solids are considered, this force balance can influence the pinning phenomena due to localized solid deformation. In the case of a thin dielectric, the direction of the electrostatic forces would favor pinning of the contact line at the soft solid surface ridge limiting the liquid advancement due to an increased vertical component of the electric forces (see discussion for Fig. 4). Thus saturation angles are higher and deviations from eqn (1) initiate at lower values of the electrowetting number. Conversely, in the case of a thick dielectric, the electrostatic forces having a pronounced horizontal component and a wider distribution favor TPL advancing, thus decreasing the contact angle. The effect of thickness becomes more pronounced as the elastic modulus, *E*, gets smaller, *i.e.* for softer materials. For high values of *E*, as Fig. 2 shows, electrowetting behavior seems to be independent of the dielectric thickness, when 7 μm ≤ *d* ≤ 50 μm.

For the softest film examined here, a deformation with a height of ≈1 μm is expected (estimated by ∼ γ/E). Interestingly, the calculation of the force acting in the vicinity of the TPL pointed out an important aspect of the subsequent stress distribution. For thin dielectric films the vertical component of the electric stress is significantly higher compared to thick ones (see Fig. 3). And this is probably attributed to the higher contact angles at saturation, observed for thin dielectrics, due to pinning of the TPL induced by the vertical electrostatic force. In summary, in accordance with previous studies,^35,38^ our computations showed that the electric field distribution could strongly affect the electrowetting performance especially for deformable solids. Thus, experimental and computational results are consistent.

## 4 Conclusions

In this work, we studied the effect of elasticity and thickness of the dielectric substrate on the contact angle saturation in electrowetting. Our experiments showed that when dielectrics are used that are thinner than 20 μm and softer than *E* < 100 kPa, then saturation sets in for lower applied voltages and higher contact angles, than in the case of rigid dielectrics. To support our experiments, we performed continuum level computations by accounting for the coupled electric field and liquid interface shape of entire droplets. Of particular interest was the electric field and electric stress distribution near the three-phase contact line (TPL).

We found a dependence of the electric stress distribution on the dielectric thickness. For thick dielectrics, electric stresses point mostly in the horizontal direction, whereas for thin dielectrics the electric stresses point mostly in the vertical direction. Vertical stresses in the vicinity of the TPL favor vertical deformation when soft substrates are used. We, then, speculated that this deformation enhances contact line pinning, thus favors electrowetting limitation in high contact angles. Considerably more work will be needed to determine whether the latter argument is correct, requiring a comprehensive study of the ridge shape under high voltages using confocal microscopy.

The insights gained from this study may also be of assistance to recent research in soft electrowetting, highlighting the effect of elasticity on the apparent contact angle as well as its correlation with dielectric thickness. As far as their viscoelasticity and their effect on spreading dynamics are concerned, soft substrates could be potentially used in applications such as lab-on-a-chip devices. Regarding that electrowetting on soft thin substrates can lead to a much lower contact angle modification which restricts their performance, thickness must be seriously considered. The dielectric thickness is an important geometrical feature that must be taken into consideration when a soft substrate is used in an EWOD configuration.

## Author contributions

I. E. M. performed the experimental work and prepared the manuscript, D. G. S. performed the computational work, N. T. C. supervised the computational work and contributed to the writing of the manuscript, P. P. prepared and characterized the samples and the corresponding parts of the manuscript and A. G. P. supervised the team and revised the manuscript.

## Conflicts of interest

The authors declare no conflict of interest.

## Supplementary Material

SM-017-D0SM02281K-s001
