# Supplementary material for: Impact of substrate elasticity on contact angle saturation in electrowetting
Source: Soft Matter. 2021 Mar 10;17(16):4335–41. doi: 10.1039/d0sm02281k (PMC8110038; doi:10.1039/d0sm02281k)
Supplement: SM-017-D0SM02281K-s001 [file SM-017-D0SM02281K-s001.pdf]

Electronic Supplementary Material (ESI) for Soft Matter.
This journal is © The Royal Society of Chemistry 2021

Impact of Substrate Elasticity on Contact Angle Saturation in Electrowetting

Supplementary Material

Ioannis E. Markodimitrakis[a], Dionysios G. Sema[a], Nikolaos T. Chamakos[a], Periklis Papadopoulos[b,c] and A. G. Papathanasiou[a]

[a]School of Chemical Engineering, National Technical University of Athens, 15780, Greece

[b]Department of Physics, University of Ioannina, Greece

[c]Institute of Materials Science and Computing, University Research Center of Ioannina, Greece

Sample preparation

In the present work, electrowetting experiments were performed on PDMS dielectric films with an elastic modulus of 1.7 MPa, 500 kPa, 100 kPa and 40 kPa for three dielectric thickness cases, 50 μm, 20 μm and 7 μm. The samples were prepared in accordance with the procedure discussed in “Material and methods”. The crosslinked polydimethylsiloxane (PDMS) films were assembled on 20x20 mm^2 glass slides covered with a conductive indium-tin oxide (ITO) layer, as shown in Figure 1. To make crosslinked PMDS with varying elastic modulus the Sylgard 184 silicone elastomer kit (Sigma-Aldrich) was used. By varying the proportion of the monomer to the curing agent the mechanical properties of the films were controlled. The preparation conditions as well as the mechanical properties as obtained from [1], [2] are presented in Table 1.

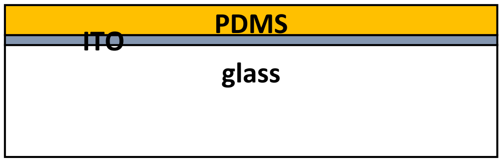

Figure 1: Glass slides with an indium-tin oxide (ITO) layer covered with PDMS.

Table 1: Preparation conditions, thickness, and mechanical properties of PDMS films.

Sample no.	monomer : curing agent mass ratio	spin coating conditions	PDMS thickness (μm)	Young's modulus (MPa) [1][2]
1	10:1	2000 rpm / 30 s	50 ± 3	1.7
2	10:1	4000 rpm / 30 s	20 ± 2	1.7
3	10:1	5000 rpm / 5 min	7 ± 1	1.7
4	20:1	4000 rpm / 30 s	20 ± 2	0.50
5	20:1	5000 rpm / 5 min	7 ± 1	0.50

6	30:1	4000 rpm / 30 s	20 ± 2	0.10
7	30:1	5000 rpm / 5 min	7 ± 1	0.10
8	40:1	2000 rpm / 30 s	50 ± 3	0.04
9	40:1	4000 rpm / 30 s	20 ± 2	0.04
10	40:1	5000 rpm / 5 min	7 ± 1	0.04

Sample characterization

Before conducting electrowetting experiments, the samples were characterized by their static contact angle and contact angle hysteresis as described in "Experimental setup & methods". The measurements were carried out on the commercial goniometer/tensiometer ramé-hart (model 100) mounted with an automated dispensing system. The contact angle hysteresis was estimated by the method of adding/removing volume. In particular, a droplet of 7 µl was deposited via the micro syringe of the dispensing system and rested on the sample for 20 s. With the micro syringe immersed in the drop, its volume was then increased in steps of 0.1 µL/s. The advancing contact angle was measured after each volume increment as the volume increased up to 12 µL. At this point, the droplet was kept at constant volume for further 20 s. By decreasing the volume of the droplet with the same rate to a final volume of 2 µL, the receding contact angle was obtained. At the final volume of 2 µL, the contact angle was measured for

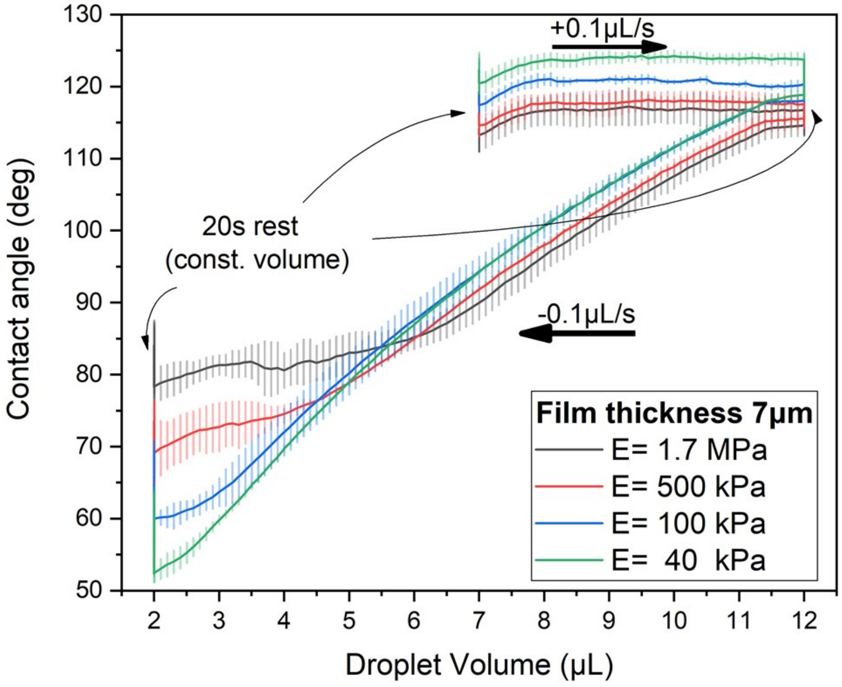

Figure 2: Advancing and receding contact angle measurement of a sessile water droplet on 7 µm thick elastomer PDMS films of variant elasticity.

another 20 s.

Table 2: Static contact angles, macroscopic advancing and receding angles of deionized water on elastomer PDMS films of variant elasticity.

monomer : curing agent mass ratio	Thickness (μm)	Static CA (deg)	Advancing CA (deg)	Receding CA (deg)	Hysteresis $\Delta\theta=\theta_{adv}-\theta_{rec}$
10:1	50 ± 3	114.1 ± 0.9	117.1±1.1	77.9±1.4	**39.2**
	20 ± 2	113.1 ± 1.6	116.3±1.9	75.1±1.8	**41.2**
	7 ± 1	113.9 ± 1.8	116.8±0.9	78.9±1.1	**37.9**
20:1	20 ± 2	116.3 ± 2.1	119.1±1.8	63.7±2.1	**55.4**
	7± 1	116.4 ± 1.6	118.1±1.2	68.5±1.8	**49.6**
30:1	20 ± 2	120.9 ± 1.6	123.8±1.8	52.6±1.8	**71.2**
	7 ± 1	119.1 ± 1.5	121.1±1.1	60.1±1.3	**61**
40:1	50 ± 3	123.2 ± 1.4	124.8±1.7	50.1±1.8	**74.7**
	20 ± 2	123.4 ± 1.3	126.1±2	45.8±2.1	**80.3**
	7 ± 1	122.3 ± 2.4	124.3±1.9	53.6±1.9	**70.7**

The higher apparent contact angles and contact angle hysteresis observed on samples with a lower Young's modulus is solely an effect of substrate viscoelasticity. The surface chemistry is hardly affected by mixing of the two components of the Sylgard 184 kit. The first one, the "monomer", is actually a linear poly(dimethyl siloxane) molecule, vinyl-terminated. One can find the structure here [3]. The curing agent cross-links with these linear PDMS chains. The exact composition is proprietary, but according to the Material Safety Datasheet (MSDS) the curing agent contains: (i) Dimethyl, Methylhydrogen Siloxane, Trimethylsiloxy-terminated, (ii) Dimethyl Siloxane, Dimethylvinylsiloxy-terminated, (iii) Dimethylvinylated and trimethylated silica nanoparticles, (iv) <1% of solvent. Thus, the first two components (>70% of total mass) are siloxanes and have very similar structure to the linear PDMS "monomer" and the silica nanoparticles also have a similar surface coating. So, the surface chemistry is not affected by adding the curing agent, which was anyway less than 10% in all cases. What changes between samples is the number of methylene "bridges" between the linear PDMS molecules that affect bulk viscoelastic properties [4]. The crosslinking reaction can be found here [5]. The final product is a PDMS network. The varying mixing ratio has otherwise a minimal effect on density, well below 1%, so the surface tension is expected to change at most by this amount, which cannot have any measurable effects.

Many previous experimental studies [6-7] have reported that the contact angle of milimetre-sized droplets on soft substrates increases with decreasing elastic modulus. The deposition of a liquid droplet on a solid surface that is deformable, results in the formation of a wetting ridge in the vicinity of the three-phase contact line (TPCL). The water surface essentially "pulls" upward the soft substrate. The apparent contact angle that is measured from the horizontal plane may, thus, differ from the Young's angle [8].

The height and aspect ratio of the ridge increase with decreasing elastomer modulus. The so-called static contact angle is usually very close to the advancing contact angle of the liquid. So,

according to the Gibbs relation (see, e.g., [9]) the apparent contact angle (measured from the horizontal plane) increases, because the PDMS-air interface to the right of the ridge has a negative slope. This slope increases, in absolute value, with decreasing modulus, so the apparent contact angle increases.

Electrowetting experiments

In this section, the data obtained from the electrowetting experiments are once again presented. In order to identify the effect of thickness on samples of the same elasticity, the apparent contact angle with respect to the applied voltage and the corresponding electrowetting number were plotted. The measurements are summarized in Figures 3 and 4.

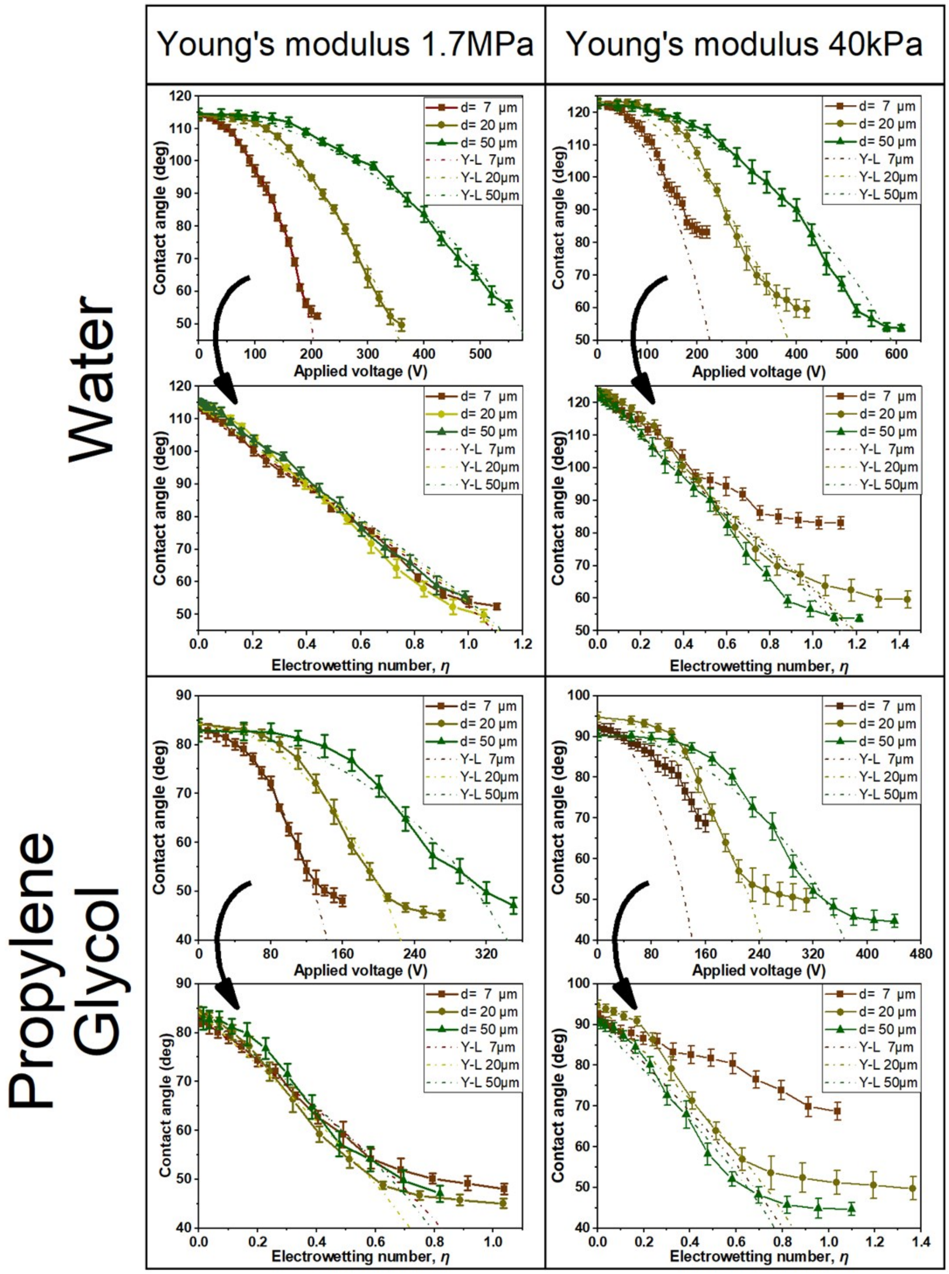

Figure 3: Apparent contact angle as a function of the applied voltage and the corresponding electrowetting number for 10 μL water and propylene glycol droplets. The experimental data are compared to the Young-Lippmann equation for each case (plotted with dash-dot lines). (first column) The electrowetting behaviour on the hardest dielectrics examined i.e. 1.7 MPa. Samples of varying thickness exhibit the same apparent contact angle at saturation. No deviation from the Y-L prediction is observed. The experimental data collapse to the same mastecurve for both liquids tested. (second column) The electrowetting behaviour on the softest dielectrics examined i.e. 40 kPa. Contact angle saturation sets in at larger contact angles with decreasing thickness. It is also clear that there is a significant deviation from the Young-Lippmann prediction for the thinnest, 7 μm thick, dielectric for both liquids.

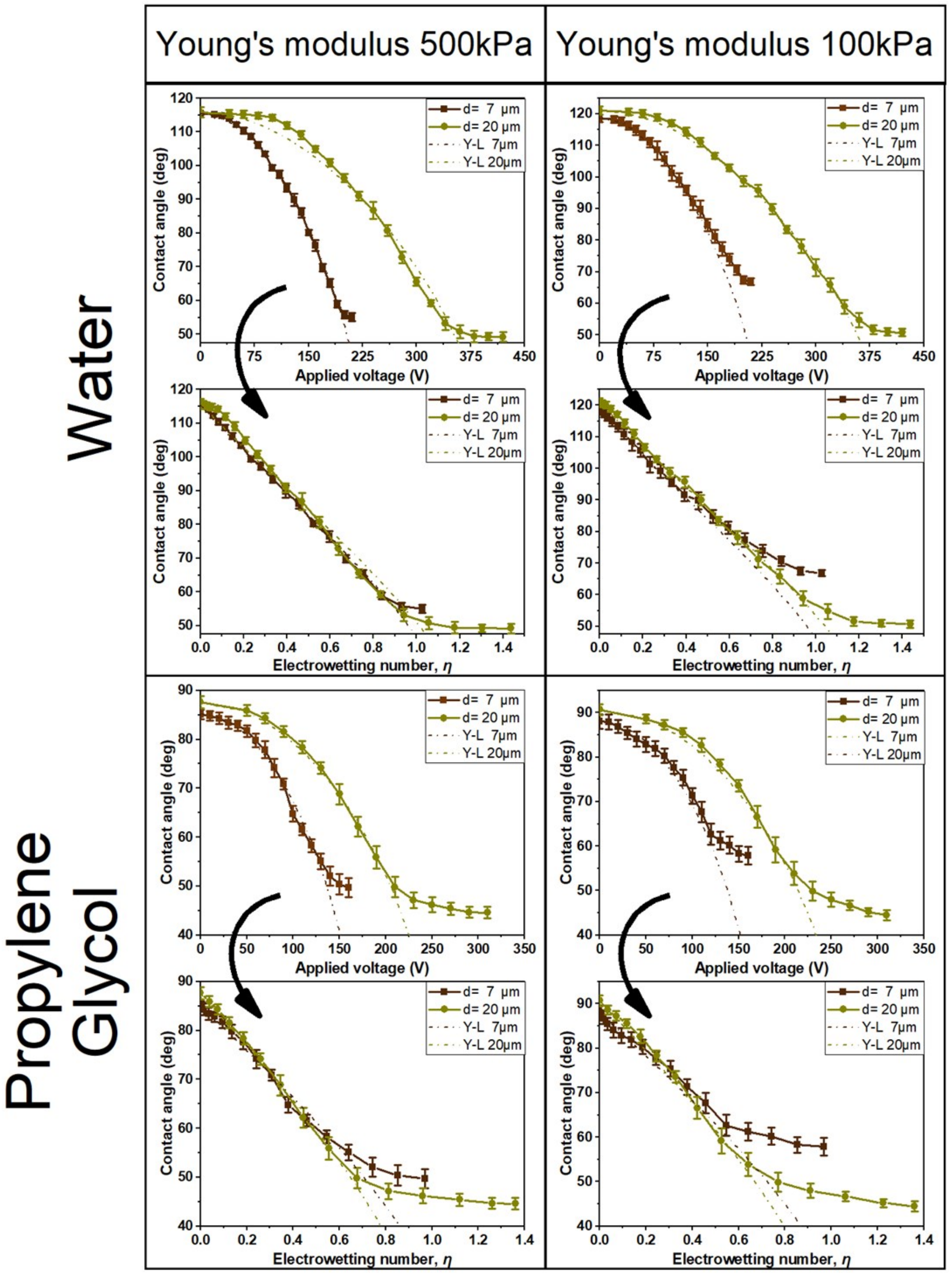

Figure 4: Apparent contact angle as a function of the applied voltage and the corresponding electrowetting number for 10 μL water and propylene glycol droplets. The experimental data are compared to the Young-Lippmann equation for each case (plotted with dash-dot lines). (first column) The electrowetting behaviour on films with a Young's modulus of 500 kPa. Contact angle saturation sets in at similar contact angles for samples of varying thickness. For both liquids tested, the experimental data collapse to the same mastecurve.There is no significant deviation from the Y-L equation. (second column) The electrowetting behaviour on films with a Young's modulus of 100 kPa. Contact angle saturation is observed at greater contact angles for relatively thin (7 μm) dielectrics. There is a slight deviation from the Young-Lippmann prediction for the thinnest, 7 μm thick, dielectric which is evident at η > 0.55.

Electrowetting modeling – Disjoining pressure with electrostatics model

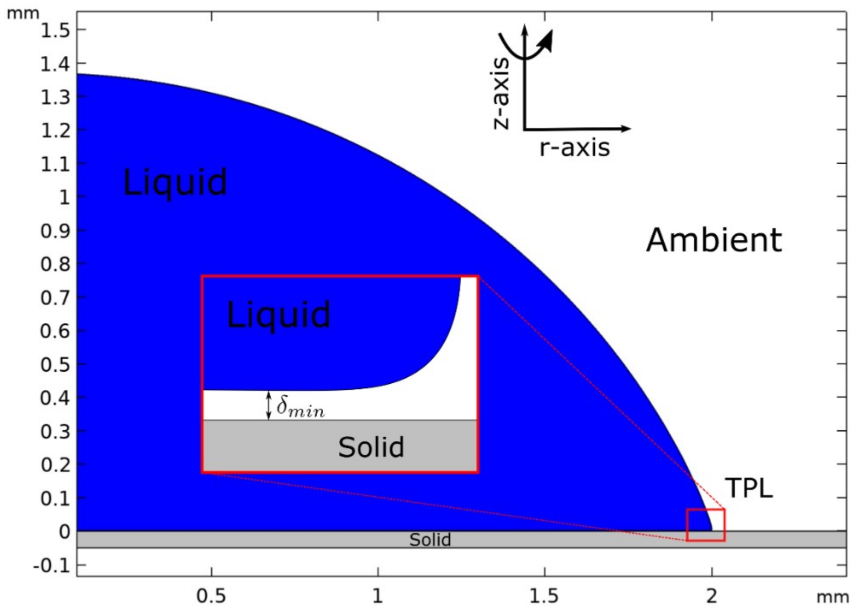

Figure 5: Schematic of the electrowetting setup of a 2D axisymmetric droplet resting on a planar substrate with a dielectric layer of thickness, d.

We perform numerical simulations to study the wetting behavior of a 2D axisymmetric droplet on a rigid flat dielectric layer with thickness, d_{el}, when applying an electric potential, V (see figure 5). The dynamics of the liquid droplet are governed by the Navier-Stokes equations, which describe the conservation of mass and momentum, and are given by:

$$\rho\left(\frac{\partial u}{\partial t} + u \cdot \nabla u\right) = \nabla \cdot T + \rho g,$$

$$\rho \nabla \cdot u = 0,$$

where $u = (u_r, u_z)$ is the fluid velocity field, g denotes the gravitation field and $T = \mu[\nabla u + (\nabla u)^T]$ the total stress tensor. The droplet is considered to be an incompressible and conductive Newtonian fluid, with density, ρ, viscosity, μ and surface tension, γ_{LA} (liquid-ambient interfacial tension). In accordance to your previous work, we treat the liquid-gas and liquid-solid interfaces in a unified manner that form the liquid-ambient interface around the droplet. Therefore, the solution of the Navier-Stokes equations is determined by imposing a single boundary condition along the liquid-ambient interface.

$$n \cdot T \cdot n = \gamma_{LA} C - p^{LS} + p_{el},$$

where C is the local curvature. The above equation represents the normal stress component and includes the liquid-solid and electrostatic interactions. The liquid-solid interactions are lumped in a disjoining (Derjaguin) pressure term, p^{LS}:

$$p^{LS} = \frac{\gamma_{LA}}{R_0}\omega^{LS}\left[\left(\frac{\sigma}{\frac{\delta}{R_0}+\varepsilon}\right)^{C_1} - \left(\frac{\sigma}{\frac{\delta}{R_0}+\varepsilon}\right)^{C_2}\right],$$

where R_0 is the initial radius of the droplet, $\sigma, \varepsilon, C_1, C_2$ are parameters in the potential, $\delta(r,z)$ is the Euclidean distance of the droplet from the nearest wall and ω^{LS} is the depth of the potential, which sets the Young's contact angle implicitly according to Dupré's approach [10]. The distance, $\delta_{min} = R_0(\sigma - \varepsilon)$, corresponds to the intermediate layer thickness of the solid-liquid interface and occurs when the attractive and repulsive forces balance out – by solving for $p^{LS}(\delta_{min}) = 0$. In equation (normal stress boundary condition), $p_{el} = \frac{\varepsilon_o E^2}{2}$, where ε_o is the vacuum permittivity and E is the electric field magnitude, represents the contribution of the electric potential to the wetting state of the droplet. The electric field, $E = -\nabla V$, is calculated by solving Gauss' law:

$$\nabla \cdot \left(\varepsilon_o \varepsilon_r \nabla V\right) = 0,$$

where the relative permittivity, ε_r, is assumed to have a value of 1 for the ambient phase (air) and 2.4 for the solid dielectric.

Finally, the moving interface is captured by imposing the following kinematic boundary condition along the liquid-ambient interface:

$$\left(u_{mesh} - u\right) \cdot n = 0.$$

The 2D axisymmetric model is implemented using the COMSOL Multiphysics® software package.

References:

[1] E. Gutierrez and A. Groisman, PLoS ONE 6, e25534 (2011).
[2] I.D. Johnston, D.K. McCluskey, C.K.L. Tan and M.C. Tracey, Journal of Micromechanics and Microengineering, 2014, 24, 035017.
[3] Sigma-Aldrich. (2021, March 05). Product Chemical Synthesis – Sylgard 184. Retrieved from https://www.sigmaaldrich.com/catalog/product/aldrich/761036
[4] B. Andreotti, and J.H. Snoeijer, Annual Review of Fluid Mechanics, 2020, 52, 285-308.
[5] HTS Resources. (2021, March 05). Crosslinking Reaction. Retrieved from https://www.htsresources.com/miccell_manual_html_5953f8c5.png
[6] L. Chen, G. K. Auernhammer, and E. Bonaccurso, Soft Matter, 2011, 7(19), 9084-9089.
[7] L. Chen, E. Bonaccurso, and M. E. Shanahan, Langmuir, 2013, 29(6), 1893-1898.
[8] L. Chen, E. Bonaccurso, T. Gambaryan-Roisman, V. Starov, N. Koursari and Y. Zhao, Current Opinion in Colloid & Interface Science, 2018, 36, 46–57.
[9] C. W. Extrand and Sung In Moon, Langmuir, 2008, 24 (17), 9470-9473.
[10] N. T. Chamakos, D. G. Sema and A.G. Papathanasiou, Archives of Computational Methods in Engineering, 2020, 1-20.
